# Gestational lipid profile as an early marker of metabolic syndrome in later life: a population-based prospective cohort study

**DOI:** 10.1186/s12916-020-01868-4

**Published:** 2020-12-23

**Authors:** Maria C. Adank, Laura Benschop, Sophia P. van Streun, Anna M. Smak Gregoor, Monique T. Mulder, Eric A. P. Steegers, Sarah Schalekamp-Timmermans, Jeanine E. Roeters van Lennep

**Affiliations:** 1 Department of Obstetrics and Gynaecology, Erasmus MC, University Medical Centre Rotterdam, Rotterdam, the Netherlands; 2 Generation R Study Group, Erasmus MC, University Medical Centre Rotterdam, Rotterdam, The Netherlands; 3 Department of General Medicine, Erasmus MC, University Medical Centre Rotterdam, PO Box 2040, 3000 CA Rotterdam, The Netherlands

**Keywords:** Pregnancy, Metabolic syndrome, Lipoproteins, HDL, Triglycerides, Cholesterol, LDL, Placental syndrome

## Abstract

**Background:**

In pregnancy lipid levels increase with gestation resembling an atherogenic lipid profile. Currently it is unclear whether gestational lipid levels are associated with an adverse cardiovascular risk profile later in life. The aim of this study is to assess the association between gestational lipid levels and lipid levels and prevalence of the metabolic syndrome (MS) six years after pregnancy.

**Methods:**

In plasma of 3510 women from the Generation R Study; a prospective population-based cohort, we measured lipid levels (total cholesterol, triglycerides and high-density lipoprotein cholesterol [HDL-c]), and low-density lipoprotein cholesterol (LDL-c), remnant cholesterol and non-HDL-c were calculated in early pregnancy (median 13.2 weeks, 90% range [10.5 to 17.1]) and six years after pregnancy (median 6.5 years, 90% range [6.2 to 7.8]). MS was assessed six years after pregnancy according to the NCEP/ATP3 criteria. We also examined the influence of pregnancy complications on these associations.

**Results:**

Gestational lipid levels were positively associated with corresponding lipid levels six years after pregnancy, independent of pregnancy complications. Six years after pregnancy the prevalence of MS was 10.0%; the prevalence was higher for women with a previous placental syndrome (13.5%). Gestational triglycerides and remnant cholesterol in the highest quartile and HDL-c in the lowest quartile were associated with the highest risk for future MS, independent of smoking and body mass index.

**Conclusions:**

Gestational lipid levels provide an insight in the future cardiovascular risk profile of women in later life. Monitoring and lifestyle intervention could be indicated in women with an unfavorable gestational lipid profile to optimize timely cardiovascular risk prevention.

**Supplementary Information:**

The online version contains supplementary material available at 10.1186/s12916-020-01868-4.

## Background

Pregnancy has been proposed as a natural ‘stress test’ to predict the risk of cardiovascular disease (CVD) later in life [1]. The long-term effect of high blood pressure during pregnancy has been well established [2–6]. Women who develop a hypertensive disorder of pregnancy such as gestational hypertension or pre-eclampsia have a 2–8 times higher risk of developing chronic hypertension, metabolic syndrome and CVD later in life compared to women with an uncomplicated pregnancy [3, 7–11]. Less is known about the long term cardiovascular effect of gestational lipid levels.

In pregnancy lipid levels rise, leading to 30% higher levels of cholesterol, triglycerides and LDL-c at the end of the 3rd term [12–15]. The gestational lipid profile therefore shows resemblance to an atherogenic lipid profile. After delivery, lipid levels in general normalize to pre-pregnancy levels within three to four months [16–18].

Previously it has been shown that women with an adverse gestational lipid profile have an increased risk of gestational hypertension and pre-eclampsia [19, 20]. The other way around, women with a hypertensive disorder of pregnancy also show a more atherogenic lipid profile six years after pregnancy than women with a previous normotensive pregnancy which may contribute to their increased CVD risk in later life [21].

Currently, it is unknown whether the gestational lipid profile, similar to gestational blood pressure, may provide a glimpse of the future cardiovascular health. Moreover it is unknown if body mass index (BMI) is the main driver for the association between gestational lipid levels and lipid levels years after pregnancy. Therefore, the aim of this study is to assess the association between gestational lipid levels with lipid levels and prevalence of the metabolic syndrome six years after pregnancy and to explore to what extent these associate with smoking and BMI. Moreover, as women with pregnancy complications may have a more adverse lipid profile we assessed the incidence of metabolic syndrome and repeated all analyses in women with and without a pregnancy complicated by the placental syndrome (pre-eclampsia, a child born small-for-gestational age and spontaneous preterm birth).

## Methods

### Design and study population

**Fig. 1:**
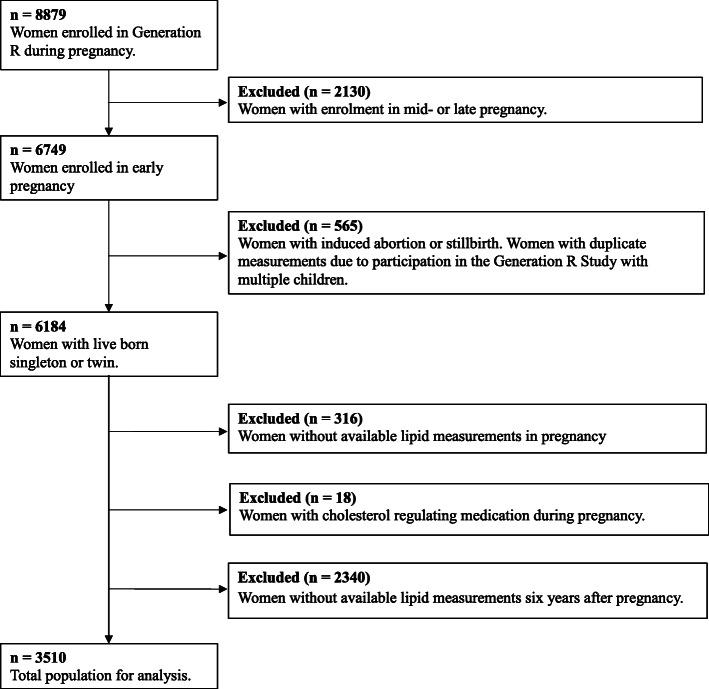
Flowchart

This study was embedded in the Generation R study, an ongoing population-based prospective cohort study from early pregnancy onwards in Rotterdam, the Netherlands [22]. Ethical approval for this study was obtained by the Medical Ethics Committee of the Erasmus University Medical Centre (Erasmus MC), Rotterdam, the Netherlands (MEC-2007-413). For this study, we included women with a live born singleton or twin. We excluded women without available lipid measurements during and/or six years after pregnancy, and women taking lipid lowering medication at intake (Fig. 1). Written informed consent was obtained from all participants. Additional file 2 contains a Strengthening the Reporting of Observational Studies in Epidemiology (STROBE) statement for the current study [23].

### Exposure: lipid levels in early pregnancy

Non-fasting blood samples were obtained via venous puncture in early pregnancy (median 13.2 weeks, 90% range [10.5 to 17.1]).

### Outcome: lipid levels six years after pregnancy

Non-fasting blood samples were obtained via venous puncture six years after pregnancy (median 6.5 years, 90% range [6.2 to 7.8]). All samples were taken by trained research nurses and stored at the research facility at room temperature for a maximum of three hours, after which they were sent to a dedicated laboratory facility [22, 24]. Glucose levels, total cholesterol, triglycerides and HDL-c concentrations were measured with the Vital Scientific (Merck) Selectra E Chemistry Analyzer. LDL-c, remnant cholesterol and non-HDL-c were calculated [25]. Details of processing procedures and lipid calculations have been described previously [20, 21, 24].

### Outcome: metabolic syndrome

Systolic blood pressure and diastolic blood pressure were measured six years after pregnancy using a validated Omron 907 automated digital oscillometric sphygmomanometer (OMRON Healthcare Europe BV, Hoofddorp, the Netherlands) [26]. According to the NCEP, ATP III criteria, metabolic syndrome was defined as having three or more of the following parameters: waist circumference (> 88 cm); triglyceride levels (≥ 150 mg/dL / ≥ 1.70 mmol/L) or drug treatment for elevated triglycerides; HDL-c (< 50 mg/dL / < 1.30 mmol/L for women); blood pressure (≥ 130 / ≥ 85 mmHg) or drug treatment for hypertension, and fasting glucose (≥ 110 mg/dL) or the use of glucose lowering medication [27]. Non-fasting glucose measured six years after pregnancy ≥ 7.8 mmol/L was used as a proxy for fasting glucose according to the cut-offs of the International Diabetes Federation. Waist circumference was measured ten years after pregnancy (median 9.7 years, 90% range [9.5 to 10.3]) at the minimum circumference between the iliac crest and the rib cage and was used as a proxy for waist circumference six years after pregnancy.

### Placental syndrome

Placental syndrome was defined as having pre-eclampsia, a child born small-for-gestational age (SGA) or a spontaneous preterm birth (sPTB) in the index pregnancy. We obtained information on clinically diagnosed pre-eclampsia from medical records that were cross-checked with the original hospital charts [28]. Pre-eclampsia was defined, using the ISSHP criteria that were in effect at the time of the study, as the development of SBP ≥ 140 mmHg and/or a DBP ≥ 90 mmHg with new-onset proteinuria in a random urine sample and no evidence of a urinary tract infection [29]. Midwife and hospital registries provided information on gestational age at birth, birth weight and child’s sex. SGA was defined as a child with a birth weight below the 10th percentile adjusted for gestational age and sex of the child. We defined sPTB as the spontaneous onset of labour before 37 weeks of gestation.

### Lifestyle factors and anthropometrics

Information on characteristics during pregnancy including maternal age, ethnicity, educational level, parity, smoking and information on folic acid supplementation was obtained by questionnaires. Six years after pregnancy, questionnaires were used to obtain information on cholesterol and glucose lowering medication. Height (cm) and weight (kg) were obtained in early pregnancy (median 13.2 weeks of gestational age, 90% range [10.5 to 17.1] weeks). Height was measured at the research facility without shoes and weight was measured without heavy clothing. Body mass Index (BMI) (kg/m^2^) was calculated based on measured weight and height. Pre-pregnancy BMI was obtained from questionnaires. The pre-pregnancy weight and the weight measured at enrolment in early pregnancy were highly correlated (Pearson’s correlation coefficient 0.96 [*P*-value < .001]). Therefore, pre-pregnancy weight was used in the analyses.

### Statistical analyses

**Fig. 2:**
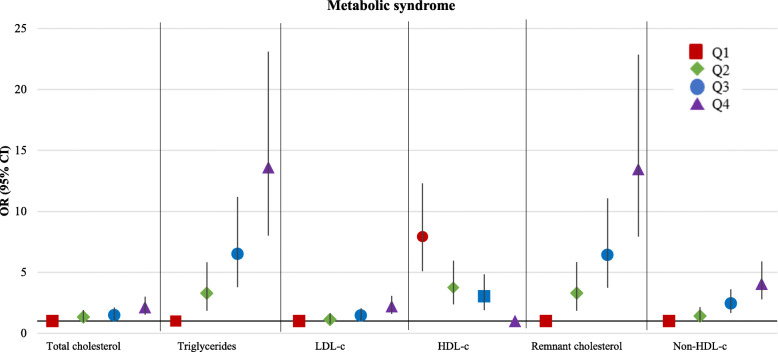
Association of maternal lipid levels in early pregnancy in quartiles with metabolic syndrome six years after pregnancy (n = 3510). Values are odds ratios (95% confidence interval) derived from multiple logistic regression analyses. Presented data are adjusted for maternal age, gestational age at blood sampling, ethnicity, educational level, parity, smoking, and folic acid supplementation. Abbreviations: OR, odds ratio; CI, confidence interval; Q, quartile; LDL-c, low-density lipoprotein cholesterol; HDL-c, high-density lipoprotein cholesterol

**Fig. 3:**
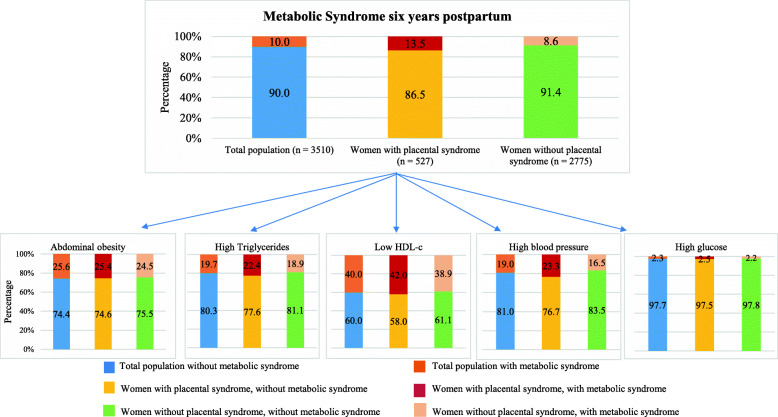
Characteristics of participants with and without metabolic syndrome. Metabolic syndrome according to the ATP III Diagnostic criteria. Metabolic syndrome is stratified by its components. For each component, the valid percentages of participants with and without metabolic syndrome are presented. Abbreviations: HDL-c, high-density lipoprotein cholesterol

We examined pregnancy and follow-up characteristics for all women (Table 1). We examined the distribution of lipid levels in early pregnancy for all women with and without the metabolic syndrome six years after pregnancy (Table 2). The presented *P-*values are the result of Student *t* test for variables with a normal distribution and Mann-Whitney U test for variables with a skewed distribution. Triglyceride and remnant cholesterol levels were log transformed to achieve a normal distribution. To enable comparison of effect estimates, we constructed SD-scores (SDS) of all lipid levels. We examined the incidence of metabolic syndrome and stratified this by its components (Fig. 3). We imputed missing values in confounders that were used for linear and logistic regression analyses. We used the Markov Chain Monte Carlo multiple imputation procedures to reduce potential bias attributable to missing data [30]. Data were analyzed in each set separately, and pooled estimates from the five imputed data sets were used to report the effect estimates and their 95% confidence interval. For the multiple imputation procedure we performed ten iterations [31]. In this study 5.0% of women had missing information on educational level, 1.3% on ethnicity, 0.5% on parity, 9.3% on smoking, 21.3% on folic acid supplementation and 16.5% on pre-pregnancy BMI. To relate lipid levels in early pregnancy to lipid levels and metabolic syndrome six years after pregnancy we performed linear and logistic regression analyses (Table 3 and Additional file 1: Table S1). To assess whether the association of lipid levels in early pregnancy and metabolic syndrome was stronger in women with higher concentrations of lipid levels (total cholesterol, triglycerides, LDL-c, remnant cholesterol and non-HDL-c) in early pregnancy and respectively lower levels of HDL-c, lipid levels in early pregnancy were categorized as quartiles and subsequently used as a categorical measure (Fig. 2). Confounders that were included in the regression models were selected based on their associations with the exposure and outcomes of interest and based on previous studies. The selected confounders included: maternal age at intake, gestational age at blood sampling, educational level, ethnicity, parity, smoking, and folic acid supplementation. For the association of gestational lipid levels with lipid levels six years after pregnancy, pre-pregnancy BMI was additionally added to the regression models. In attempt to exclude the effect of placental syndromes, we examined the incidence of metabolic syndrome in this subgroup of women and repeated all the analyses excluding women with a placental syndrome in their index pregnancy (Fig. 3). A nonresponse analysis was conducted by comparing the characteristics of women included in this study (*n* = 3510) to women without available lipid levels six years after pregnancy (*n* = 2340) (Additional file 1: Table S2). Statistical analyses were performed using the IBM Statistical Package of Social Sciences version 24.0 for Windows (SPSS Inc., Chicago, IL).

**Table 1:** Characteristics of the total population (*n* = 3510)

Outcomes in pregnancy	Mean (SD) or Median (90% range)
Age mother, years	30.3 (4.8)
Gestational age at blood sampling, weeks	13.2 (10.5 to 17.1)
Non-European ethnicity, n (%)	1254 (35.7)
Low educational level, n (%)	317 (9.0)
Nulliparous, n (%)	2226 (63.4)
Pre-pregnancy BMI, kg/m^2^	22.7 (18.8 to 32.0)
Smoking during pregnancy, n (%)	947 (27.0)
No folic acid supplementation, n (%)	836 (23.8)
SBP in early pregnancy, mmHg	116.0 (12.4)
DBP in early pregnancy, mmHg	68.5 (9.6)
Total cholesterol, mmol/L	4.84 (0.88)
Triglycerides, mmol/L	1.26 (0.72 to 2.33)
LDL-c, mmol/L	2.43 (0.73)
HDL-c, mmol/L	1.79 (0.35)
Remnant cholesterol, mmol/L	0.57 (0.33 to 1.06)
Non-HDL-c, mmol/L	3.05 (0.84)
Glucose, mmol/L	4.38 (0.85)
Gestational age at birth, weeks	40.1 (36.9 to 42.1)
Birth weight, g	3414 (558)
Small-for-gestational age, n (%)	350 (10.0)
Spontaneous preterm birth, n (%)	125 (3.6)
Pre-eclampsia, n (%)	80 (2.4)
**Six years after pregnancy**	
Interval time, years	6.5 (6.2 to 7.8)
Age mother, years	37.0 (4.8)
Maternal BMI, kg/m^2^	24.4 (19.7 to 35.0)
Total cholesterol, mmol/L	4.87 (0.89)
Triglycerides, mmol/L	1.14 (0.61 to 2.51)
LDL-c, mmol/L	2.91 (0.78)
HDL-c, mmol/L	1.37 (0.33)
Remnant cholesterol, mmol/L	0.52 (0.28 to 1.13)
Non-HDL-c, mmol/L	3.50 (0.89)
Glucose, mmol/L	5.47 (0.99)
Waist circumference, cm	82.8 (11.0)
SBP, mmHg	119.2 (12.7)
DBP, mmHg	70.6 (9.9)
Metabolic syndrome, n (%)	350 (10.0)

Data are presented as mean (SD), median (90% range) or number of subjects (valid percentage) depending on normal or skewed distributions. Covariates are imputed. Waist circumference was measured ten years after pregnancy. Abbreviations: SBP, systolic blood pressure; DBP, diastolic blood pressure; LDL-c, Low-density lipoprotein cholesterol; HDL-c, High-density lipoprotein cholesterol; BMI, body mass index

**Table 2:** Lipid distribution in early pregnancy for women with and without the metabolic syndrome six years after pregnancy (*n* = 3510)

	Without metabolic syndrome *n* = 3160	With metabolic syndrome *n* = 350	***P***-value
Total cholesterol, mmol/L	4.81 (0.87)	5.09 (0.90)	< 0.001
Triglycerides, mmol/L	1.22 (0.71 to 2.19)	1.66 (1.00 to 3.09)	< 0.001
LDL-c, mmol/L	2.41 (0.72)	2.68 (0.78)	< 0.001
HDL-c, mmol/L	1.81 (0.34)	1.58 (0.33)	< 0.001
Remnant cholesterol, mmol/L	0.55 (0.32 to 1.00)	0.75 (0.45 to 1.37)	< 0.001
Non-HDL-c, mmol/L	3.00 (0.82)	3.50 (0.88)	< 0.001

Data are presented as mean (SD) or as median (90% range) depending on normal or skewed distributions. Differences between groups are tested through a Students *t* test or Mann Whitney U test. Abbreviations: LDL-c, low-density lipoprotein cholesterol; HDL-c, high-density lipoprotein cholesterol

**Table 3:** Association of maternal lipid levels in early pregnancy with metabolic syndrome six years after pregnancy (n = 3510)

	Without metabolic syndrome *n* = 3160	With metabolic syndrome OR (95% CI) *n* = 350	***P***-value
Total cholesterol, SDS	*Reference*	1.30 (1.16 to 1.45)	< 0.001
Triglycerides, SDS	*Reference*	2.43 (2.14 to 2.75)	< 0.001
LDL-c, SDS	*Reference*	1.36 (1.22 to 1.51)	< 0.001
HDL-c, SDS	*Reference*	0.51 (0.46 to 0.58)	< 0.001
Remnant cholesterol, SDS	*Reference*	2.43 (2.14 to 2.76)	< 0.001
Non-HDL-c, SDS	*Reference*	1.66 (1.49 to 1.86)	< 0.001

Values are odds ratios (95% confidence interval) derived from multiple logistic regression analyses. Basic model: adjusted for maternal age, gestational age at blood sampling, ethnicity, educational level, parity, smoking and folic acid supplementation. Abbreviations: SDS, SD-scores; CI, confidence interval; OR, odds ratio; LDL-c, low-density lipoprotein cholesterol; HDL-c, high-density lipoprotein cholesterol

## Results

This study included a total of 3510 women (Table 1). Women were on average 30.3 years old and 13.2 weeks pregnant when they entered the study. 64.3% had a European ethnicity, 47.1% was highly educated, 63.4% was nulliparous at intake and they had on average a pre-pregnancy BMI of 22.7 kg/m^2^.

### Early gestational lipid levels and lipid levels six years postpartum

Total cholesterol, triglycerides, LDL-c, HDL-c, remnant cholesterol and non-HDL-c levels were positively associated with their corresponding lipid level six years after pregnancy (Additional file 1: Table S1). These associations were independent of pre-pregnancy BMI.

### Early gestational lipid levels and metabolic syndrome six years postpartum

Six years after pregnancy, 350 (10.0%) women were classified as having metabolic syndrome. 47.1% of women with (gestational) diabetes mellitus or women with glucose lowering medication in pregnancy developed metabolic syndrome six years later. The prevalence of metabolic syndrome in women with diabetes mellitus and/or glucose or cholesterol lowering medication at follow-up was 68.2%. Compared to women without metabolic syndrome, women with metabolic syndrome six years after pregnancy had a more atherogenic plasma lipid profile in early pregnancy, significant for all analyzed lipids (*P-*value < .001) (Table 2).

Table 3 shows the association between maternal lipid levels in early pregnancy with metabolic syndrome six years after pregnancy. Women with higher levels of total cholesterol, triglycerides, LDL-c, remnant cholesterol and non-HDL-c levels in early pregnancy were more at risk of developing metabolic syndrome six years after pregnancy. These associations remained statistically significant after adjustment for confounders. HDL-c was negatively and independently associated with metabolic syndrome.

Figure 2 shows that compared to total cholesterol and LDL-c levels in the lower quartile, those in the highest quartile were associated with an increased risk of metabolic syndrome (OR 2.13, 95% CI [1.52 to 2.99] and OR 2.20, 95% CI [1.58 to 3.06], respectively). Compared to HDL-c levels in the highest quartile, those in the lower quartiles were associated with an increased risk of metabolic syndrome, with the highest risk for women in the lowest quartile (OR 7.91, 95% CI [5.09 to 12.29]). Compared to triglycerides and remnant cholesterol levels in the lowest quartile, early pregnancy levels in the second, third and fourth quartile were associated with an increased risk of metabolic syndrome (*P-*value for trend analysis < .001). Women with triglyceride and remnant cholesterol levels in the highest quartile had the highest risk of metabolic syndrome (OR 13.61, 95% CI [8.02 to 23.10] and OR 13.46, 95% CI [7.93 to 22.85], respectively). Compared to non-HDL-c levels in the lowest quartile, early pregnancy non-HDL-c levels in the third and fourth quartile were associated with an increased risk of metabolic syndrome, with the highest risk for women in the highest quartile (OR 4.05, 95% CI [2.79 to 5.88]).

### Metabolic syndrome and its components

The most common feature of metabolic syndrome was low HDL-c (40.0%) followed by abdominal obesity (25.6%), high triglycerides (19.7%), a high blood pressure (19.0%), and high glucose levels (2.3%) (Fig. 3).

### Women without placental syndrome vs women with placental syndrome

Of the 2775 women without a placental syndrome in their index pregnancy, 239 (8.6%) were classified as having metabolic syndrome six years after their index pregnancy. The prevalence of individual MS components were comparable to those of the total population: low-HDL-c 38.9%, abdominal obesity 24.5%, high triglycerides 18.9%, high blood pressure 16.5%, and high glucose levels 2.2% (Fig. 3).

Of the 527 women with a placental syndrome in their index pregnancy, 71 (13.5%) were classified as having metabolic syndrome six years after pregnancy. Their most common feature of metabolic syndrome was also low HDL-c (42.0%) followed by abdominal obesity (25.4%), a high blood pressure (23.3%), high triglycerides (22.4%), and high glucose levels (2.5%) (Fig. 3).

Women with a placental syndrome had a higher risk (OR 1.55, 95% CI [1.15 to 2.07]) to develop metabolic syndrome six years after pregnancy compared to women without a placental syndrome.

In addition, the results of our regression analyses were not influenced by placental syndrome since the results were comparable when the analyses were repeated in a subset of women without placental syndrome in their index pregnancy (*n* = 2775) (data not shown).

## Discussion

This study shows that lipid levels in early pregnancy are positively associated with lipid levels and the prevalence of metabolic syndrome six years after pregnancy. These associations remained significant after correction for smoking and BMI.

Previous studies on lipid levels measured in pregnancy and later in life are mostly limited to measurements up to one year postpartum [16, 17, 32]. These studies show that lipid levels initially decrease in pregnancy followed by a gradual increase and peak at the end of the third trimester. Lipid levels decline to plateau at four months after the delivery [18]. Postpartum total cholesterol levels and LDL-c levels remain somewhat but are significantly higher than the levels measured before pregnancy. However, these studies do not report on the association of gestational lipid levels with lipid levels after pregnancy. A previous study performed within the Generation R study population found that lipid levels in early pregnancy increase moderately with advancing gestational age in the time-frame when lipids in this study were measured [20]. Results from this study show that lipid levels measured in early pregnancy may already be indicative for lipid levels six years later, independent of smoking and BMI. Since early pregnancy lipid levels are also associated with adverse pregnancy outcomes and blood pressure during and after pregnancy [20], measurement of lipid levels in early pregnancy may improve short and long-term health of women.

The prevalence of metabolic syndrome in our relatively young and highly educated population of women was 10.0%. Data on prevalence of metabolic syndrome in young women is scarce. Notably it is much lower than the prevalence of 22–28% of metabolic syndrome observed in American women aged 30–49 years described in the National Health and Nutrition Examination Survey (NHANES) [33]. A possible explanation may be that their mean BMI was higher and that their educational level was lower which is associated with an increased risk of metabolic syndrome [34].

Previous studies assessed the prevalence of metabolic syndrome in women who experienced hypertensive disorders of pregnancy, however almost all of these studies lacked a control group. Within these populations some found that women with early-onset pre-eclampsia had a higher risk of developing metabolic syndrome [8], whereas others did not find such an association [35]. A Canadian study, consisting of 217 women, found that the prevalence of metabolic syndrome after one and three years after pregnancy was 18.2% and 21.9% in women with previous pre-eclampsia and 6.8 and 6.4% in women without pre-eclampsia [36]. The mean BMI in that study was higher compared to that in our study (28.7 kg/m2 and 26.3 kg/m2 in women with and without pre-eclampsia compared to 24.4 kg/m2 in our population), which may account for the higher incidence of MS.

We hypothesized that BMI would be the main risk factor driving the association between gestational lipids and the lipid profile six years after pregnancy. However, these associations remained significant also after adjusting for pre-pregnancy BMI. Lipid levels are partially determined by lifestyle factors, but also by genetics [37, 38]. A recent study found that in a healthy population of women LDL-c levels ≥ 99th percentile were in 45.5% of the cases explained by unfavorable genotypes or mutations associated with hypercholesterolemia [37]. Several other studies also found that genetic background has a large influence on lipid concentrations which may be the reason why these associations remain significant after adjusting for BMI [38–40]. This is interesting since it suggests that lipid levels have a certain level of stability; independent of lifestyle factors. Therefore, unfavorable lipid levels may be an early marker for future cardiovascular risk. In our population the majority of women remained in the same quartile in pregnancy and six years after pregnancy (data not shown), supporting this hypothesis. As shown in previous studies, women with a placental syndrome have an increased risk of metabolic syndrome and CVD later in life [41]. Therefore, we performed the same analyses in a subset of women without placental syndromes. This did not affect our results. We therefore hypothesize that our results may be explained by genetic inheritance and to a lesser extent by lifestyle or pregnancy-related factors. Unfortunately, the nature of our study did not allow further investigation of this genetic contribution.

In young, fertile women monitoring of lipid levels may be relevant due to the association with CVD later in life. Currently, guidelines of the American Heart Association and guidelines of the European Society of Cardiology do not advice to determine gestational lipid levels [42, 43]. However, in our opinion adding lipids to the routine blood measurements in early pregnancy may provide an opportunity to early identify women with an increased CVD risk profile. This may be beneficial for timely intervention and prevention of metabolic syndrome and subsequently possible CVD in later life. Especially as women are more prone to improve lifestyle during pregnancy [44].

### Strengths and limitations

Strengths of our study are the prospective and structured data collection from early pregnancy onwards. We also have a large sample of 3510 women with blood samples six years after pregnancy available. Some limitations need to be considered. First, blood samples were obtained in a non-fasting state. However, according to the joint consensus statement from the European Atherosclerosis Society and the European Federation of Clinical Chemistry and Laboratory Medicine lipids and lipoproteins change minimally in response to normal food intake [45].

Second, pre-pregnancy BMI was self-reported. Nevertheless, pre-pregnancy BMI was strongly correlated with BMI measured in early pregnancy, which makes misclassification unlikely.

Third, waist circumference was only measured ten years after pregnancy. However, weight measured six years after pregnancy was highly correlated to weight measured ten years after pregnancy, as did BMI six and ten years after pregnancy (Pearson’s correlation coefficients, r 0.91 and 0.91, respectively [*P* < .001]). Therefore, we assumed that waist circumference ten years after pregnancy could be used as a proxy for waist circumference six years after pregnancy.

Finally, similar to other studies, the non-response six years after pregnancy may have led to selection of relatively healthy women, which may affect the generalizability of results to high-risk populations (Additional file 1: Table S2).

## Conclusion

The gestational lipid profile is associated with the lipid profile and metabolic syndrome six years after pregnancy, independent of smoking and BMI. Having a more atherogenic gestational lipid profile may act as an early risk marker for CVD later in life. Therefore, monitoring and possibly even early intervention should be indicated in women with a more atherogenic gestational lipid profile to diminish the cardiovascular burden later in life.

## Supplementary Information

**Additional file 1: Table S1.** Association of maternal lipid profile in early pregnancy with their corresponding lipid levels six years later (*n* = 3510). **Table S2.** Baseline characteristics of women with and without available lipid measurements six years after pregnancy.

**Additional file 2.** STROBE statement.

## Data Availability

Data requests can be made to the secretary of Generation R.

## Abbreviations

ATP: Adult Treatment Panel
BMI: Body mass index
CI: Confidence interval
CVD: Cardiovascular disease
DBP: Diastolic blood pressure
HDL-c: High-density lipoprotein cholesterol
LDL-c: Low-density lipoprotein cholesterol
MS: Metabolic syndrome
NCEP: National Cholesterol Education Program
OR: Odds ratio
SBP: Systolic blood pressure
SD: Standard deviation
SDS: Standard deviation scores
SGA: Small-for-gestational age
sPTB: Spontaneous preterm birth
