# Supplementary material for: Gestational lipid profile as an early marker of metabolic syndrome in later life: a population-based prospective cohort study
Source: BMC Med. 2020 Dec 23;18:394. doi: 10.1186/s12916-020-01868-4 (PMC7756942; doi:10.1186/s12916-020-01868-4)
Supplement: Supplementary file 1 — Additional file 1: Table S1. Association of maternal lipid profile in early pregnancy with their corresponding lipid levels six years later (n = 3510). Table S2. Baseline characteristics of women with and without available lipid measurements six years after pregnancy. [file 12916_2020_1868_MOESM1_ESM.docx]

**SUPPLEMENTAL MATERIAL**

**Additional file 1, Table S1.** Association of maternal lipid profile in early pregnancy with their corresponding lipid levels six years later (n = 3510).

| **Lipids in pregnancy** | **Basic model**  Beta (95% CI) | **BMI model**  Beta (95% CI) |
| --- | --- | --- |
| Total cholesterol, SDS | 0.57 (0.54 to 0.59) | 0.57 (0.54 to 0.60) |
| Triglycerides, SDS | 0.48 (0.45 to 0.51) | 0.46 (0.43 to 0.49) |
| LDL-c, SDS | 0.59 (0.56 to 0.62) | 0.59 (0.56 to 0.61) |
| HDL-c, SDS | 0.55 (0.52 to 0.58) | 0.53 (0.50 to 0.55) |
| Remnant cholesterol, SDS | 0.46 (0.43 to 0.50) | 0.44 (0.41 to 0.47) |
| Non-HDL-c, SDS | 0.59 (0.56 to 0.62) | 0.58 (0.56 to 0.61) |

Values are beta’s (95% CI) derived from multiple linear regression analyses. Basic model: adjusted for maternal age, gestational age at blood sampling, ethnicity, educational level, parity, smoking and folic acid supplementation. BMI model: basic model additionally adjusted for pre-pregnancy BMI. Abbreviations: SDS, SD-scores; CI, confidence interval; LDL-c, low-density lipoprotein cholesterol; HDL-c, high-density lipoprotein cholesterol.

**Additional file 1, Table S2.** Baseline characteristics of women with and without available lipid measurements six years after pregnancy.

| **Characteristics in pregnancy** | **No lipid measurements available six years after pregnancy**  (n = 2340) | **Lipid measurements available six years after pregnancy**  (n = 3510) | ***P-*value** |
| --- | --- | --- | --- |
| Age mother, years | 28.6 (5.2) | 30.3 (4.8) | <0.001 |
| Gestational age at blood sampling, weeks | 13.4 (10.5 to 17.4) | 13.2 (10.5 to 17.1) | 0.02 |
| Non-European ethnicity, n (%) | 1170 (50.0) | 1254 (35.7) | <0.001 |
| Low educational level, n (%) | 372 (15.9) | 317 (9.0) | <0.001 |
| Nulliparous, n (%) | 1356 (57.9) | 2226 (63.4) | <0.001 |
| Pre-pregnancy BMI, kg/m^2^ | 22.7 (18.3 to 33.0) | 22.7 (18.8 to 32.0) | 0.19 |
| Smoking during pregnancy, n (%) | 738 (31.5) | 947 (27.0) | 0.001 |
| No folic acid supplementation, n (%) | 820 (35.0) | 836 (23.8) | <0.001 |
| SBP in early pregnancy, mmHg | 115.2 (12.5) | 116.0 (12.4) | 0.02 |
| DBP in early pregnancy, mmHg | 68.1 (9.7) | 68.5 (9.6) | 0.13 |
| Gestational age at birth, weeks | 40.0 (36.3 to 42.1) | 40.1 (36.9 to 42.1) | 0.001 |
| Birth weight, g | 3362 (592) | 3414 (558) | <0.001 |
| Small-for-gestational age, n (%) | 262 (11.4) | 350 (10.0) | 0.09 |
| Spontaneous preterm birth, n (%) | 108 (4.7) | 125 (3.6) | 0.04 |
| Pre-eclampsia, n (%) | 73 (3.4) | 80 (2.4) | 0.04 |

Data are presented as mean (SD), median (90% range) or number of subjects (valid percentage) depending on normal or skewed distributions. Covariates are imputed. Abbreviations: SBP, systolic blood pressure; DBP, diastolic blood pressure; BMI, body mass index.
